# Co-targeting triple-negative breast cancer cells and endothelial cells by metronomic chemotherapy inhibits cell regrowth and migration *via* downregulation of the FAK/VEGFR2/VEGF axis and autophagy/apoptosis activation

**DOI:** 10.3389/fonc.2022.998274

**Published:** 2022-11-30

**Authors:** Arianna Scagliotti, Laura Capizzi, Marina Elena Cazzaniga, Alice Ilari, Marco De Giorgi, Nicoletta Cordani, Matteo Gallazzi, Antonino Bruno, Giuseppe Pelosi, Adriana Albini, Marialuisa Lavitrano, Emanuela Grassilli, Maria Grazia Cerrito

**Affiliations:** ^1^ School of Medicine and Surgery, University of Milano-Bicocca, Monza, Italy; ^2^ Phase 1 Research Center, Azienda Socio Sanitaria Territoriale (ASST) di Monza, Monza, Italy; ^3^ Laboratory of Immunology and General Pathology, Department of Biotechnology and Life Sciences, University of Insubria, Varese, Italy; ^4^ Laboratory of Innate Immunity, Unit of Molecular Pathology, Biochemistry and Immunology, Istituto di Ricovero e Cura a Carattere Scientifico (IRCCS) MultiMedica, Milan, Italy; ^5^ Istituto di Ricovero e Cura a Carattere Scientifico (IRCCS) MultiMedica, Milan, Italy; ^6^ Department of Oncology and Hemato-Oncology, University of Milan, Milan, Italy; ^7^ IRCCS European Institute of Oncology (IEO), Milan, Italy

**Keywords:** metronomic chemotherapy, triple negative breast cancer (TNBC), endothelial cells, angiogenesis, FAK-VEGFR2-VEGF-axis

## Abstract

High-dose standard-of-care chemotherapy is the only option for triple-negative breast cancer (TNBC) patients, which eventually die due to metastatic tumors. Recently, metronomic chemotherapy (mCHT) showed advantages in treating TNBCs leading us to investigate the anti-metastatic and anti-angiogenic potential of metronomic 5-Fluorouracil plus Vinorelbine (5-FU+VNR) on endothelial cells (ECs) and TNBCs in comparison to standard treatment (STD). We found that 10-fold lower doses of 5-FU+VNR given mCHT vs. STD inhibits cell proliferation and survival of ECs and TNBC cells. Both schedules strongly affect ECs migration and invasion, but in TNBC cells mCHT is significantly more effective than STD in impairing cell migration and invasion. The two treatments disrupt FAK/VEGFR/VEGF signaling in both ECs and TNBC cells. mCHT, and to a much lesser extent STD treatment, induces apoptosis in ECs, whereas it switches the route of cell death from apoptosis (as induced by STD) to autophagy in TNBC cells. mCHT-treated TNBCs-derived conditioned medium also strongly affects ECs’ migration, modulates different angiogenesis-associated proteins, and hampers angiogenesis in matrix sponge *in vivo*. In conclusion, mCHT administration of 5-FU+VNR is more effective than STD schedule in controlling cell proliferation/survival and migration/invasion of both ECs and TNBC cells and has a strong anti-angiogenic effect. Our data suggest that the stabilization of tumor growth observed in TNBC patients treated with mCHT therapy schedule is likely due not only to direct cytotoxic effects but also to anti-metastatic and anti-angiogenic effects.

## Introduction

Triple-negative breast cancer (TNBC) is an aggressive histological subtype of breast cancer characterized by the lack of estrogen receptor (ER), progesterone receptor (PR), and lack of amplification/overexpression of human epidermal growth factor receptor 2 (HER2). Accounting for about 12-17% of all breast carcinomas (1), TNBC is more aggressive than other breast tumors, and it often correlates with short disease-free survival (DFS) and overall survival (OS) (2, 3). Chemotherapy remains the primary therapeutic option for TNBC patients because neither endocrine therapies nor HER2-targeted agents can be used in this subtype of breast cancer. Several data have shown that TNBCs express high levels of intratumoral vascular endothelial growth factor (VEGF) (4) and display VEGF gene amplification compared to non-TNBCs (5), suggesting a strong angiogenic dependency and thus a potential sensitivity to anti-angiogenic factors. Despite numerous drugs have been approved for anti-angiogenic therapies, their success has been quite limited, providing only a short pause in tumor growth before the onset of drug resistance, thus often allowing only a modest survival benefit (6). In addition, many cancers can gain access to blood supply through vascular co-optation, thus evading the need for tumor angiogenesis (7).

Angiogenesis is a multistep process that involves different players, i.e., tumor cells, immune cells, and endothelial cells (ECs), and the balance of anti-angiogenic and pro-angiogenic stimuli is the main regulatory mechanism of the process. During cancer progression, the tumor microenvironment disrupts this balance in favor of stimuli that promote the proliferation and migration of ECs (8) which are among the principal players in angiogenesis; in fact, their responses to extracellular stimuli such as VEGF are essential during the growth of blood vessels as well as for organ growth and repair (9). Among different VEGF receptors, VEGFR2 has been identified as the principal mediator of many physiological and pathological consequences of VEGF on ECs, including proliferation, migration, survival, and permeability (10). One of the downstream signaling mediators following VEGFR2 activation is focal adhesion kinase (FAK) (11), which is crucial for ECs migration. Indeed, activated FAK is recruited to new focal adhesions where it phosphorylates paxillin thereby leading to the cytoskeletal rearrangements necessary for ECs to migrate (12). Other than in ECs, FAK also plays a role in cancer cells: it has been shown that high FAK expression in breast tumors is associated with more aggressive tumor types such as lymphovascular invasion and triple-negative phenotype (13). In addition, Pan et al. indicated FAK as a prognostic marker in patients with TNBC (14) thus suggesting that novel combinations of drugs targeting FAK may be helpful in patients that progress or fail to respond (15).

Microtubule-targeting drugs, such as taxanes, or vinca alkaloids, such as Vinorelbine (VNR), are the standard-of-care (SOC) in the therapy of TNBC (16, 17) and are administered according to the protocol indicated as standard chemotherapy (STD), where the drug is usually given at maximum tolerated doses for several cycles, thus leaving prolonged drug-free-breaks between administrations. Interestingly, the addition of Capecitabine – the orally available precursor of the cytotoxic moiety 5-fluorouracil, 5-FU - to SOC STD resulted in significant improvements in both DSF (HR 0.82, P = 0.004) and OS (HR 0.78, P = 0.004) in patients with early-stage TNBC (18) and provided remarkable results in the metastatic setting, therefore suggesting that the two agents are highly synergistic (19, 20). At variance with STD chemotherapy, metronomic chemotherapy (mCHT) refers instead to the minimum biologically effective dose of a chemotherapeutic agent given as a continuous regimen with no prolonged drug-free breaks. This schedule seems to have a direct cytotoxic effect on cancer cells and an effect on the tumor microenvironment, likely by inhibiting tumor angiogenesis (21, 22). In the early 2000s, several reports showed the anti-angiogenic activity of some anti-tumor agents when administered frequently and at low doses (23–25). In *in vivo* models of hepatocellular carcinoma cyclophosphamide given mCHT significantly reduced tumor growth and metastasis to a greater extent than the STD administration, showing anti-proliferative, anti-angiogenic and anti-metastatic proprieties of the drug (26).

Many clinical trials using a metronomic schedule are ongoing (27, 28), and so far, the results show a strong stabilization of cancer growth along with an improvement in the quality of life of cancer patients due to a reduction of the toxic side effects (29). Accordingly, a clinical study with 80 advanced breast cancer patients – including 28 TNBC patients - showed an improvement in the clinical benefit rate and progression-free survival after the metronomic administration of VNR plus Capecitabine (30).

We previously demonstrated that the mCHT administration of 5-FU plus VNR can induce apoptosis and autophagy in TNBC cells at lower doses than the STD administration (31). In the present study, we first evaluate the effects of mCHT administration of 5-FU+VNR on ECs. We determined that this schedule of treatment strongly affects cell survival and clonogenicity even at 10-fold lower doses than the STD treatment. We showed that the combination of 5-FU+VNR strongly impairs ECs migration/invasion and tube formation, as well as TNBC cell migration/invasion, *via* downregulation of the FAK/VEGFR2/VEGF circuit. We also showed that a direct cytotoxic effect -via apoptosis induction - is triggered in ECs differently from what we reported for TNBC cells (31). Finally, we also investigated the effect of conditioned medium derived from mCHT- and STD-treated TNBC cells on angiogenesis *in vitro* and *in vivo.* Conditioned medium from metronomically-treated TNBC cells has a strong anti-angiogenic effect on neovascularization *in vitro*, by affecting the expression of several angiogenesis-related proteins, and *in vivo*, by inhibiting the growth of new blood vessels.

## Materials and methods

### Cell lines

Human umbilical vein endothelial cells (HUVECs) were obtained from IRCCS MultiMedica, (Milan, Italy) and were cultured in Endothelial Growth Medium-2 (#CC-3156; EGM™-2 Medium, Lonza, Basel, Switzerland) supplemented with EGM™ SingleQuots™ Kit (#CC-4176 Lonza, Basel, Switzerland). HUVECs were maintained in culture until passage 6. Human TNBC cell lines MDA-MB-231 and BT-549 were purchased from American Type Culture Collection (ATCC, VA, USA) and cultured in DMEM and RPMI-1640 medium respectively (#BE12-604F; #BE12-702F Lonza, Basel, Switzerland) supplemented with 10% FBS (#ECS5000L Euroclone), 100 units/ml of penicillin and 100 mg/ml of streptomycin (#ECB3001_3380; Euroclone). In addition, RPMI-1640 medium was supplemented with 0,023 units/ml of insulin. Cells were routinely tested for the presence of Mycoplasma by Hoechst stain (#62249; ThermoFisher). All cells were kept in a humidified incubator with 5% CO2 at 37°C.

### Metronomic and standard protocols of treatment

HUVECs were plated at 2000 cells/well in 96-well plate previously coated with 0.25 μg/mL of Human Collagen Type I (#CC050, Millipore Merck, Darmstadt, Germany). The following day cells were treated with increasing doses of 5-FU (Fluoruracil Teva^®^, obtained from San Gerardo Hospital, Monza, Italy) and VNR (Vinorelbine Ditartrate Salt Hydrate, #V2264, Sigma-Aldrich). The following doses were used: 1, 5, 8, 10, 20, 50 μM of 5-FU and 0.1, 1, 10, 100, 1000 nM of VNR. In the mCHT protocol the drug-containing medium was replaced every 24 hours up to 96 hours. In the STD protocol drug-containing medium was replaced with drug-free medium after 4 hours of treatment, and this change was repeated every 24 hours up to 92 hours. MDA-MB-231 and BT-549 cells were treated with the respective IC_50s_ of 5-FU+VNR under mCHT or STD schedule as previously established (31) and showed in **Table 1**.

**Table 1:** The combination of 5-FU+VNR given under metronomic protocol affect the viability of HUVECs and TNBC cells in the same range of doses.

	IC_50_ 5–FU + VNR	
	STD	mCHT
**HUVEC**	25μM +42 nM	2.7μM +0.48 nM
**MDA–MB–231**	80μM +30 nM	4.5 μM +0.5 nM
**BT–549**	100μM +35 nM	4.5 μM +0.5 nM

**Figure 1:**
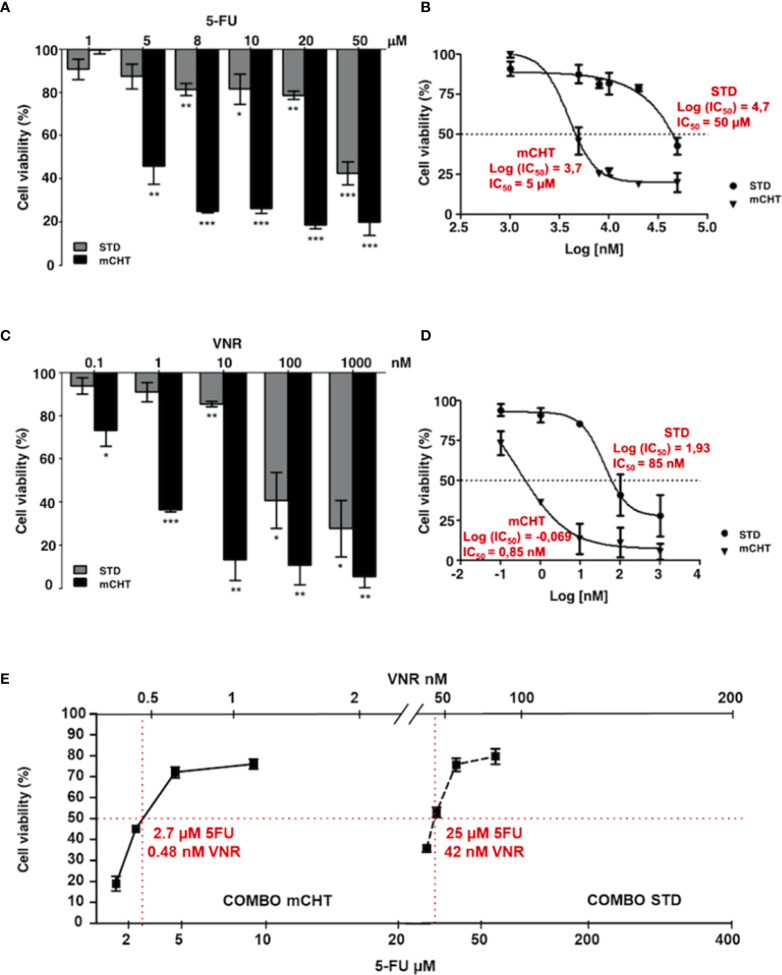
Metronomic administration of 5-FU and VNR - alone or in combination - more effectively reduces HUVECs viability, even when used at much lower concentrations than those used for standard treatment. MTT assay performed on HUVECs at the end of STD or mCHT treatment with increasing doses of 5-FU **(A)** or VNR **(C)**. Dose-response curves were used to calculate the IC_50_ values of 5-FU **(B)** or VNR **(D)** treatments. Values represent the average ± SD of three independent experiments and are expressed as the percentage of viability of treated *vs.* untreated cells *p < 0.05; **p < 0.01; ***p < 0.001. **(E)** Dose-response curve obtained from MTT assay performed at the end of STD or mCHT treatment with the following concentrations: ¼ IC_50(5-FU)_+ ¼ IC_50(VNR)_; ½ IC_50(5-FU)_+ ½ IC_50(VNR)_; IC_50(5-FU)_+ IC_50(VNR)_; 2x IC_50(5-FU)_+ 2x IC_50(VNR)_. Increasing doses of 5-FU are reported on the lower x-axis and increasing doses of VNR are reported on the upper x-axis. The simple two-point method was used to estimate the IC_50s_ (reported in red) from 2 data points that reduces cell number to 50% of the control (red lines). Values represent the average ± SD of three independent experiments and are expressed as the percentage of viability of treated *vs.* untreated cells.

Comparison of the IC_50_ values obtained in **Figure 1** treating HUVECs with 5-FU+VNR given mCHT or STD with those we previously reported for MDA-MB-231 and BT-549 cells (31).

### Cell viability assay

At the end of single and combined treatments, MTT (Methyl thiazolyl tetrazolium, #M5655, Sigma-Aldrich) was added to each well at the concentration of 1 mg/ml. After 3 hours of incubation, cells were centrifuged at 2000 rpm for 10 minutes and lysed with 100% Ethanol (#414605, Carlo Erba Reagents). The values of absorbance of the formazan salt were measured at 540 nm with Infinite 200 Pro microplate reader (Tecan Trading AG, Switzerland) and expressed as the percentage of the untreated control. IC_50_ values were calculated with Prism5 software (GraphPad Software Inc., La Jolla, California, USA). Graphs represent the average of 3 independent experiments ± standard deviation (SD).

The IC_50_ values obtained from single-drug treatments were used to design drug combination experiments as reported by Chou-Talalay (32): cells were treated with 2X IC_50_5-FU + 2X IC_50_VNR, IC_50_5-FU + IC_50_VNR, ½ IC_50_5-FU + ½ IC_50_VNR and ¼ IC_50_5-FU + ¼ IC_50_VNR.

### Colony formation assay

HUVECs, MDA-MB-231 and BT-549 cells were treated with the respective IC_50s_ of 5-FU+VNR under mCHT or STD schedule. At the end of treatments, surviving cells were trypsinized, counted and seeded at low density (1500 cells/well) in 6-wells plates. Medium was replaced every 3 days with fresh medium. After 10 days, colonies were fixed and stained with 1% crystal violet in 35% ethanol for 40 minutes. Images were acquired using G:BOX XT4 Chemiluminescence and Fluorescence Imaging System (Syngene, Cambridge, UK). The number of colonies was counted with ImageJ Software (Wayne Rasband National Institutes of Health, USA) and reported as percentage of untreated control ± SD. Images are representative of three independent experiments.

### Scratch assay

HUVECs, MDA-MB-231 and BT-549 cells were seeded at 2x10^5^ cells/well in 12-wells plates. The day after, confluent cells were scratched using a 200 μl pipette tip and photographed immediately after injury (T_0_). As a control, we also left an unscratched plate for each cell line. Cells were treated with the respective IC_50s_ following the STD and the mCHT protocol and pictures were taken 96h after treatment. In another set of experiments, unscratched plates were treated as above for 72h. Then we made a scratch, we changed the medium following the STD and mCHT schedule and we took picture. To evaluate the closing of the wound we took pictures 24h after the scratch. The change of the scratch wound size was evaluated by comparing the photos from time 0 to the 96h (the last time point) or time 72h to 96h (24h after the scratch). To obtain the measure of each scratch closure the distances between the front of the cells were measured by the ImageJ software (Wayne Rasband National Institutes of Health, USA). Images are representative of three independent experiments.

### Transwell Boyden chamber assay

The ability of cell migration and invasion were measured using transwell chambers with or without 20 μl of 7% Matrigel diluted in serum-free medium (#354262, BD Biosciences).

HUVECs, MDA-MB-231 and BT-549 cells were treated with the respective IC_50s_ of 5-FU+VNR under mCHT or STD schedule. At the end of the treatments, surviving cells were detached with 0.25% Trypsin-EDTA solution (#ECB3052, Euroclone), resuspended in serum-free medium and counted. Cells were seeded (5x10^4^ HUVECs, 4x10^4^ MDA-MB-231 and 1x10^5^ BT-549 for migration assay; 6x10^4^ cells/well for the invasion assay) in 100 μl of serum-free medium in the upper chamber of 6.5 mm Transwell^®^ chamber with 8.0 μm pores size polycarbonate membrane filters (Corning Costar, Corning, NY). In the case of migration experiments, HUVECs were seeded in wells pre-coated with Fibronectin (#11080938001, Sigma-Aldrich). Then 600 μl of medium containing 10% FBS was added the bottom of 24-well plates as chemoattractant. After overnight incubation cells migrated, and adhered onto the bottom side of the filter, were fixed with 3.6% formaldehyde (#47608, Sigma-Aldrich) for 20 min, permeabilized with 100% methanol (#34806, Sigma-Aldrich) and stained with crystal violet (#C6158, Sigma-Aldrich). Cells remained in the top surface of the membrane filter were removed with a cotton swab. Cells were counted using ImageJ Software (Wayne Rasband National Institutes of Health, USA) and reported as percentage of the untreated control ± SD. Images are representative of three independent experiments.

### Tube formation assay

HUVECs were treated with the respective IC_50s_ of 5-FU+VNR under the mCHT or STD schedule. At the end of the treatments, cells were trypsinized, counted and seeded at 2x10^4^ cells/well in 96-wells plate previously coated with 80 μl of BD Matrigel™ matrix HC phenol red-free (#3121531, BD Biosciences). After 4, 8 and 24h of incubation, images were taken and the total length of the tubes and the total meshes area were measured using ImageJ software (Wayne Rasband National Institutes of Health, USA) and reported in graphs as percentage of untreated control ± SD; the number of branching points was measured by using the automated software Wimasis WimTube (Wimasis GmbH, Munich, Germany). Images are representative of three independent experiments.

### Paracrine angiogenesis activity

Paracrine activity of TNBC cells was tested by evaluating how TNBC-derived conditioned media (CM) affected endothelial cell migration. CMs were collected from MDA-MB-231 cells treated with the IC_50_ of 5-FU +VNR under STD or mCHT schedule. The drug-containing medium of each time point of the treatments was collected, mixed, centrifuged, and used to treat HUVECs. HUVECs cultured with the conditioned medium from MDA-MB-231 pre-treated with STD and mCHT protocols are indicated, as c-STD and c-mCHT, respectively. HUVECs cultured with conditioned medium harvested from untreated MDA-MB-231 cells were used as control (c-NT). Scratch test, Transwell migration and colony formation assays were performed with the conditioned media obtained as described above.

### Western blot

HUVECs, MDA-MB-231 and BT-549 cells were treated with the respective IC_50s_ of 5-FU+VNR under the mCHT or STD schedule and lysed with RIPA buffer (HEPES 50 mM, pH 7.5, NaCl 500 mM, DTT 1 mM, EDTA 1 mM, 0.1% NP40) supplemented with 1% protease inhibitor cocktail (# P2714, Sigma-Aldrich, Milan, Italy). Protein concentration was measured by the Bradford method (Sigma Aldrich). 25μg of proteins were loaded onto 10% NuPAGE Tris-Glycine protein gels or 4-12% NuPAGE Bis-Tris protein gels (Novex, San Diego, USA) and run for 2 hours at 100 V in Tris-Glycine Running buffer or MES Running buffer (Novex, San Diego, USA). Proteins were transferred to nitrocellulose membrane (Invitrogen) by the iBlot system, followed by 1hour blocking solution with 5% BSA and incubation with the following primary antibodies: anti-BAX (1:1000, #2772 Cell Signaling), anti-BCL2 (1:1000, #4223, Cell Signaling) anti-pFAK (Y397) (1:1000, #3283 Cell Signaling), anti-FAK (1:1000, #3285 Cell Signaling), anti-VEGFR2 (1:1000, #55B11, Cells Signaling), anti-VEGF (C-1) (1:500, #65373, Santa Cruz), anti-cleaved caspase3 (1:1000, #9661, Cell Signaling), anti-LC3AB (1:1000, #4108, Cell Signaling), anti-MMP2 (1:1000, #40994, Cell Signaling), anti-TIMP1 and anti-TIMP2 (1:1000, #8946 and #5738, Cell Signaling), anti-Beclin-1 (#3738, Cell Signaling), anti-SQSTM1/p62 (#5114, Cell Signaling), anti-βactin (1:5000, #4967, GeneTex). After three washes with 0.05% PBS Tween, membranes were incubated at room temperature for 1 h with appropriate secondary antibody diluted in 5% nonfat dry milk in TBST. After three washing with 0.05% PBS-Tween, membranes were incubated with “Pierce™ ECL Western Blotting Substrate” (#32106, ThermoFisher) for 5 minutes, and images were acquired using G:BOX XT4 Chemiluminescence and Fluorescence Imaging System (Syngene, Cambridge, UK).

### Human angiogenesis array kit/proteome profiler

To analyze the soluble factors involved in the angiogenesis process, a RayBio^®^ C-Series Human Angiogenesis Antibody Array C1000 (RayBiotech) was used according to the manufacturer’s instructions. This kit can detect 43 proteins involved in angiogenesis and invasiveness thanks to the antibodies spotted in duplicate onto nitrocellulose membranes. Briefly, at the end of the experiment (96 hours), the conditioned medium of MDA-MB-231 and BT-549 cells untreated or treated with STD and mCHT protocols were collected, centrifugated and stored at -20°C until further use. Membranes were incubated with blocking buffer for 30 minutes at room temperature and then incubated with conditioned medium overnight at 4°C on a rocking platform. After washing the membranes with the appropriate buffers, a biotinylated antibody cocktail was added to the well and incubated for 2 hours at room temperature. Following the washing steps, HRP-Streptavidin solution was added onto the membrane, incubated for 2 hours at room temperature and then detection buffer was added and quickly visualized afterward. The chemiluminescent signal was acquired using G:BOX XT4 Chemiluminescence and Fluorescence Imaging System (Syngene, Cambridge, UK). The signal was then quantified with ImageJ Software (Wayne Rasband National Institutes of Health, USA) and reported as the percentage of untreated control.

### Generation of conditioned media/supernatants for the *in vivo* experiments

Cell supernatants were generated by treating MDA-MB-231 and BT-549 cells, with the respective IC_50s_ of 5-FU+VNR under mCHT or STD schedule. Twenty-four hours before the end of the experiment, the medium was changed with serum-free culture media. At 96h, conditioned media were collected, filtered to eliminate possible residual dead cells and debris, then concentrated using Millipore concentricons (Millipore), with 3KDa pores. Concentrated media were quantified, for their protein contents, using the Bradford reagent (#B6916, Sigma Aldrich). Aliquots of 50 μg of protein were prepared and immediately stored at -80°C until use for *in vivo* experiments.

### *In vivo* Ultimatrix sponge angiogenesis assay

The effect of mCHT on angiogenesis *in vivo* was tested using the Matrigel plug assay (33, 34). Briefly, 600 μl of 10mg/mL of unpolymerized liquid UltiMatrix (Reduced Growth Factor Basement Membrane Extract; #BME001, Biotechne), was mixed with 50 μg of total protein content concentrated as described above. 600μl of the generated mixtures were subcutaneously injected into the flanks of 6- to 8-week-old C57/BL6 male mice (Charles River, Italy). All animals were housed in a conventional animal facility with 12h light/dark cycles and fed *ad libitum.* Manipulations of animals were in accordance with the Italian and European Community guidelines (D.L. 2711/92 N°116; 86/609EEC Directive), the 3 R s declaration, and approved by the institutional ethics committee. Groups of 4-6 mice were used for each treatment. Four days following injection, the gel plugs were recovered and divided into 2 parts. One half was formalin-fixed, paraffin-embedded to generate paraffin blocks processed for histological analysis; the other half from BT-549 supernatant was minced and diluted in water to measure the hemoglobin content with a Drabkin’s Reagent Kit (#D5941, Sigma).

### Immunohistochemistry analysis of UltiMatrix sponges

All the processing for the immunohistochemistry analysis on the UltiMatrix sponges were performed by the Unit of Pathological Anatomy, IRCCS MultiMedica, Milan, Italy, by routinely system on an automated immunostainer (BenchMark ULTRA IHC/*in situ* hybridization System, Ventana-Roche Group, Basel, Switzerland). Haematoxylin and Eosin-stained (H&E) sections were used to acquire micrographs, at 20x and 40x of magnification. The histological examination of the vascularity intensity of each treatment independently was assessed by more than 2 blinded observers using H&E-stained slides and classified as weak positive (+), positive (++) and strongly positive (+++).

### Statistical analysis

Data are showed as means ± standard deviation (SD) of three independent experiments. The significance of results was determined with the Student’s t-test. Values with *p<0.05* are considered statistically significant. * means *p <0.05*, ** means p < *0.01* and *** means *p <0.001*.

## Results

### Metronomic administration of 5-FU+VNR is more effective than the standard administration in reducing HUVECs viability

To investigate the antiproliferative effect of STD and mCHT administration of 5-FU+VNR on HUVECs, we treated cells with increasing doses of 5-FU or VNR for 4 hours (STD) or repeated the addition of the drugs each 24 hours up to 96 hours (mCHT), as described in Materials and Methods. At the end of the treatments, we evaluated cell viability compared to not treated (NT) control cells, by MTT assay. When HUVECs were exposed to the STD schedule, a modest but significant effect was observed at lower concentrations and the highest inhibition was obtained only at the highest concentrations of the drugs tested (50μM for 5-FU and 1000 nM for VNR) (**Figures 1A, C**).

Contrarily, a strong and very significant effect on cell viability was already observed at lower concentrations (5μM for 5-FU and 1nM for VNR) when HUVECs were exposed to mCHT administration of the single drugs (**Figures 1A, C**). Indeed, compared to the STD administration of each drug, the IC_50_ of mCHT administration was about 10-fold lower for 5-FU (5μM *vs*. 50μM), and 100-fold lower for VNR (0.85nM *vs*. 85nM), as indicated by the dose-response curves in **Figures 1B, D**. Notably, the strong effects achieved by lower doses of chemotherapy are particularly relevant from the clinical point of view, since lowering drugs’ dose means less toxic side effects for patients.

Next, we performed MTT assays to evaluate the effects on cell viability of both drugs given in combination (5-FU+VNR) on HUVECs. We found that the mCHT administration of 5-FU+VNR more significantly impaired cell viability as compared to the STD treatment (**Figure 1E**), with IC_50s_ of 2.7μM 5-FU+0.48nM VNR and 25μM 5-FU+42nM VNR, respectively (**Table 1**). In other terms, 9-fold less 5-FU and 87-fold less VNR are required in mCHT *vs.* STD treatment to achieve 50% inhibition of HUVECs’ cell viability. Therefore, also in the case of ECs mCHT administration allows to significantly reduce the drugs dose compared to STD treatment, in line with what we previously reported for TNBC cell lines (31) and in **Table 1**.

Altogether these data indicate that metronomic administration of 5-FU+VNR strongly decreases HUVECs’ cell viability at concentrations significantly lower than those used for STD treatment.

### Metronomic combination of 5-FU+VNR suppresses colony formation ability of both HUVECs and TNBC cells

**Figure 2:**
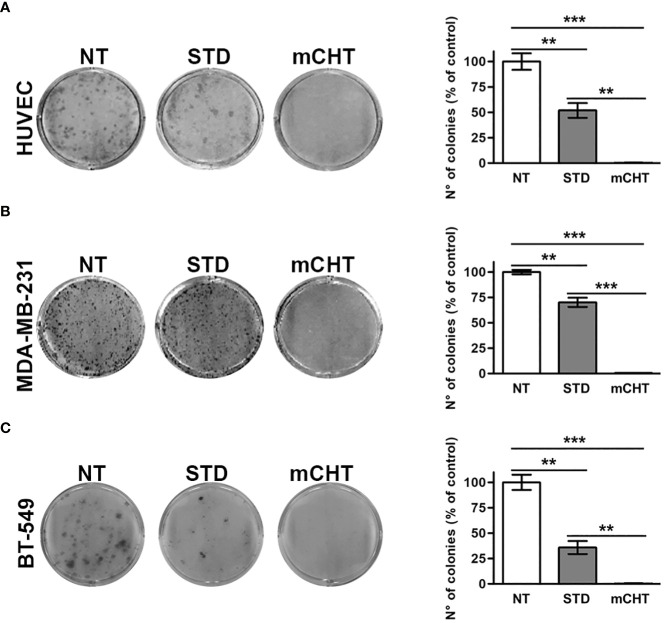
Metronomic administration of 5-FU+VNR is more effective than standard treatment in reducing HUVECs and TNBC cells colony formation. Representative images of colony formation assays stained 10 days after the end of STD or mCHT combination treatments of HUVECs **(A)**, MDA-MB-231 cells **(B)** and BT-549 **(C)**. Colonies were stained with crystal violet and counted. On the right of each panel number: number of colonies grown after treatments quantified as a percentage of untreated controls. Values represent the average ± SD of three independent experiments. **p < 0.01; ***p < 0.001.

Tumor relapses often occur after STD because of the proliferation of survived cancer cells and the restoration of damaged tumor vessels. To determine whether HUVECs and TNBC cells retain the capability to proliferate after mCHT or STD treatment we exposed the cells to the combination of 5-FU and VNR - each given at the IC_50_ concentration – and, at the end of the incubation time, we replaced the medium with drug-free complete medium allowing survived cells to grow for 10 days, at the end of which we measured colony formation. Compared to NT cells, HUVECs’ capability to form colonies was significantly reduced to half by 5-FU+VNR given STD whereas it was completely suppressed by the mCHT administration as shown in **Figure 2A**. Similarly, 5-FU+VNR-treated MDA-MB-231 cells’ ability to form colonies was significantly reduced to 70% under STD protocol, whereas it was completely abolished under mCHT schedule as indicated in **Figure 2B**. BT-549 cells - another TNBC cell line, characterized by a different mutational background (**Supplementary Table S1**)- appeared to be more sensitive to STD treatment given that the ability to form colonies was reduced to 36%; however, the therapeutic advantage of the mCHT schedule was evident since no colonies at all outgrew under this protocol as reported in **Figure 2C**.

These findings indicate that all the cell types tested are extremely sensitive to the metronomic combination and ECs are more sensitive than TNBC cells to 5FU+VNR given as STD.

### Metronomic combination of 5-FU+VNR strongly reduces cell migration and invasion of both HUVECs and TNBC cells

**Figure 3:**
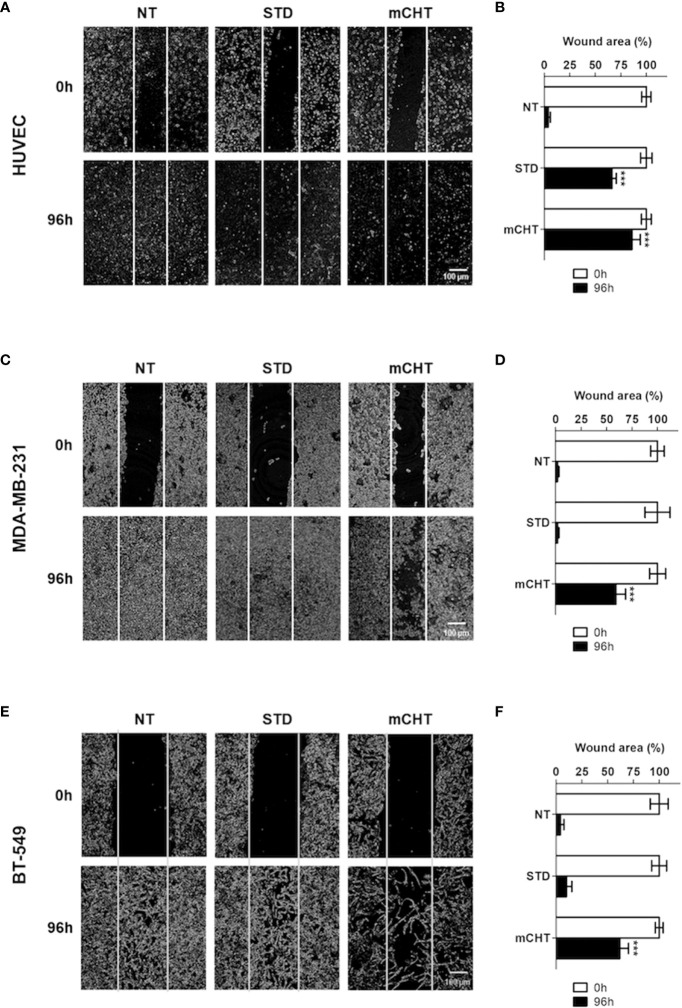
Metronomic administration of 5-FU+VNR is more efficient than standard protocol in inhibiting wound closure of HUVECs and TNBC cells. Representative images of scratch tests performed on HUVECs **(A)** MDA-MB-231 cells **(C)** and BT-549 **(E)** before (0h) and 96h after STD or mCHT treatment with 5-FU+VNR. The area of the still open wound after 96h is quantified as a percentage of the initial scratch **(B, D, F)**. Values represent the average ± SD of three independent experiments. ***p < 0.001.

Cell migration is a critical process in tumor progression for both new vessel formation and metastasis spread. Therefore, we investigated cell migration by the scratch test, performed on HUVECs and TNBC cells and evaluated after 96h of treatment with the respective IC_50s_ of 5-FU+VNR given under the STD or mCHT schedule (**Figure 3**). We found that the migratory ability of HUVECs was significantly more reduced by the administration of 5-FU+VNR under mCHT *vs* STD protocols. Indeed, about 85% *vs* 66% of the scratched areas were still open at the end of mCHT and STD administration of 5-FU+VNR, respectively, as shown in **Figures 3A, B**. In contrast, MDA-MB-231 cells’ migratory ability was remarkably reduced only under mCHT schedule being 60% of the scratched area still open at the end of the experiment, whereas it was almost unaffected by the STD treatment where only 2% of the initial scratch was still measurable (**Figures 3C, D**). Similar results were obtained on BT-549 cells where 62% of the wound was still opened under mCHT treatment compared to 10% upon STD treatment (**Figures 3E, F**).

Given that in a period of treatment as long as 96 hours additional factors, others from the migratory capability - such as the cytotoxicity-induced death of treated cells or the proliferative capability of surviving cells - could affect the closure of the wound, we evaluated the re-population of the scratched area also in a shorter time frame. Therefore, we treated the TNBC cells for 96h with the two different protocols and performed the scratch 24h before the end of the experiment (i.e., at 72h), in order to exclude that the closure of the wound might be due to an increase in the number of cells ensuing from a round of proliferation. Remarkably, the results obtained also in this setting (**Supplementary Figure S1**), resemble to those observed in the previous experiment, thus reinforcing the conclusion that the migratory capability of both ECs and TNBC cells is strongly and more significantly affected by mCHT treatment than by the STD treatment.

**Figure 4:**
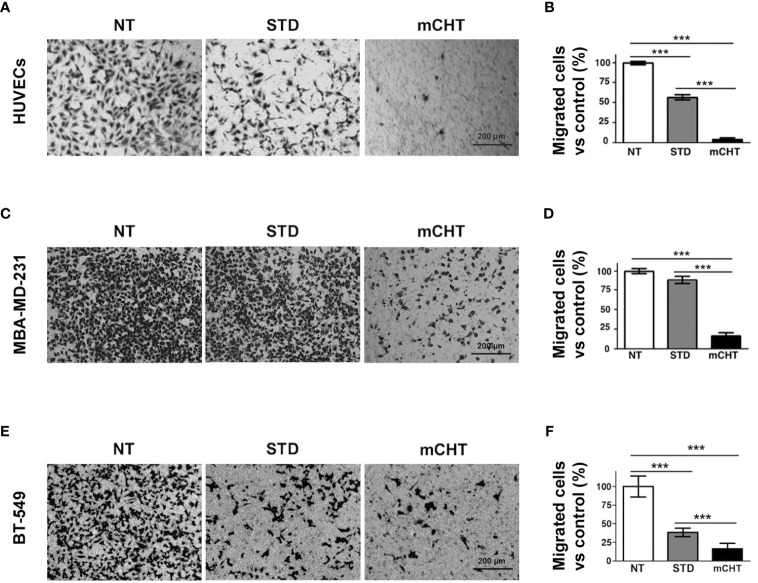
Metronomic treatment with 5-FU+VNR suppresses HUVECs and TNBC cell migration more efficiently than standard administration. Representative images of the Transwell assays on HUVECs **(A)**, MDA-MB-231 cells **(C)** and BT-549 **(E)** treated for 96h under the STD or mCHT protocol and performed as described in Material and Methods. Migrated cells were stained with crystal violet, counted, and graphically expressed as a percentage of the untreated control **(B, D, F)**.Values represent the average ± SD of three independent experiments. **p < 0.01; ***p < 0.001.

Next, we examined the migratory capability of HUVECs and TNBC cells under mCHT and STD treatments by using the Transwell assay system. Migrated cells were marked off by crystal violet staining and then counted by Image J, as shown in **Figure 4**. Notably, we found that the number of HUVECs migrated across the membrane was almost zeroed by 5-FU+VNR given mCHT (3% *vs.* NT) whereas one-fifth of the STD-treated HUVECs was still able to migrate (20.3% *vs*. NT) (**Figures 4A, B**). Importantly, the migration of MDA-MB-231 cells was strongly suppressed only when the drug combination was given as mCHT; in fact, compared to the untreated control, the percentage of migrated cells was 16% after mCHT treatment *vs.* 88% after STD (**Figures 4C, D**). Interestingly, the strong negative effect on BT-549’s migratory capability exerted by the drug combination given mCHT was very similar to that we observed for HUVECs and MDA-MB-231 cells, being only 16% of the treated cells able to traverse the membrane. Similarly, in the case of the BT-549 cells the anti-migration action of 5-FU+VNR administered STD was milder since almost two-fifths (39%) of the treated cells migrated across the membrane (**Figures 4E, F**).

**Figure 5:**
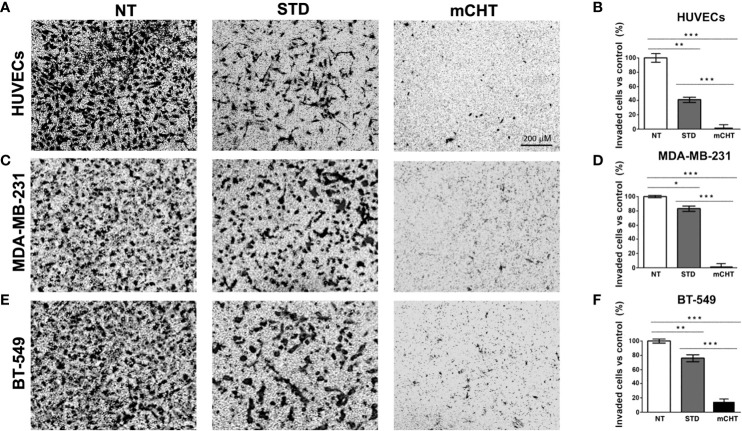
Metronomic treatment with 5-FU+VNR inhibits HUVECs and TNBC cell invasion more efficiently than standard administration. Representative images of Transwell assay on HUVECs **(A)**, MDA-MB-231 cells **(C)** and BT-549 **(E)** treated for 96h under the STD or mCHT protocol and performed as described in Material and Methods. Cells that invaded through the Matrigel‐coated membrane were stained with crystal violet, counted, and graphically expressed as a percentage of the untreated control **(B, D, F)**. Values represents the average ± SD of three independent experiments. *p < 0.05; **p < 0.01; ***p < 0.001.

To further characterize how cell migration can be affected by the 5-FU+VNR treatment we performed an invasion where we added a Matrigel layer on the top of the inserts of the Boyden chamber (**Figures 5A, C, E**). Under these conditions, a significantly stronger inhibitory effect of the mCHT compared to the STD treatment was observed for all cell lines. In detail, only 5% of HUVECs were able to invade the matrix after mCHT exposure compared to 40% after STD administration (**Figure 5B**). Notably, around 80% of TNBC cells retained the ability to invade the matrix after STD treatment whereas only 4% and 15% of MDA-MB-231 and BT-549, respectively, invaded the lower chamber upon mCHT administration (**Figures 5D, F**). These results suggest that STD chemotherapy only mildly affects whereas mCHT administration strongly impairs the invasion ability of TNBC cells.

**Figure 6:**
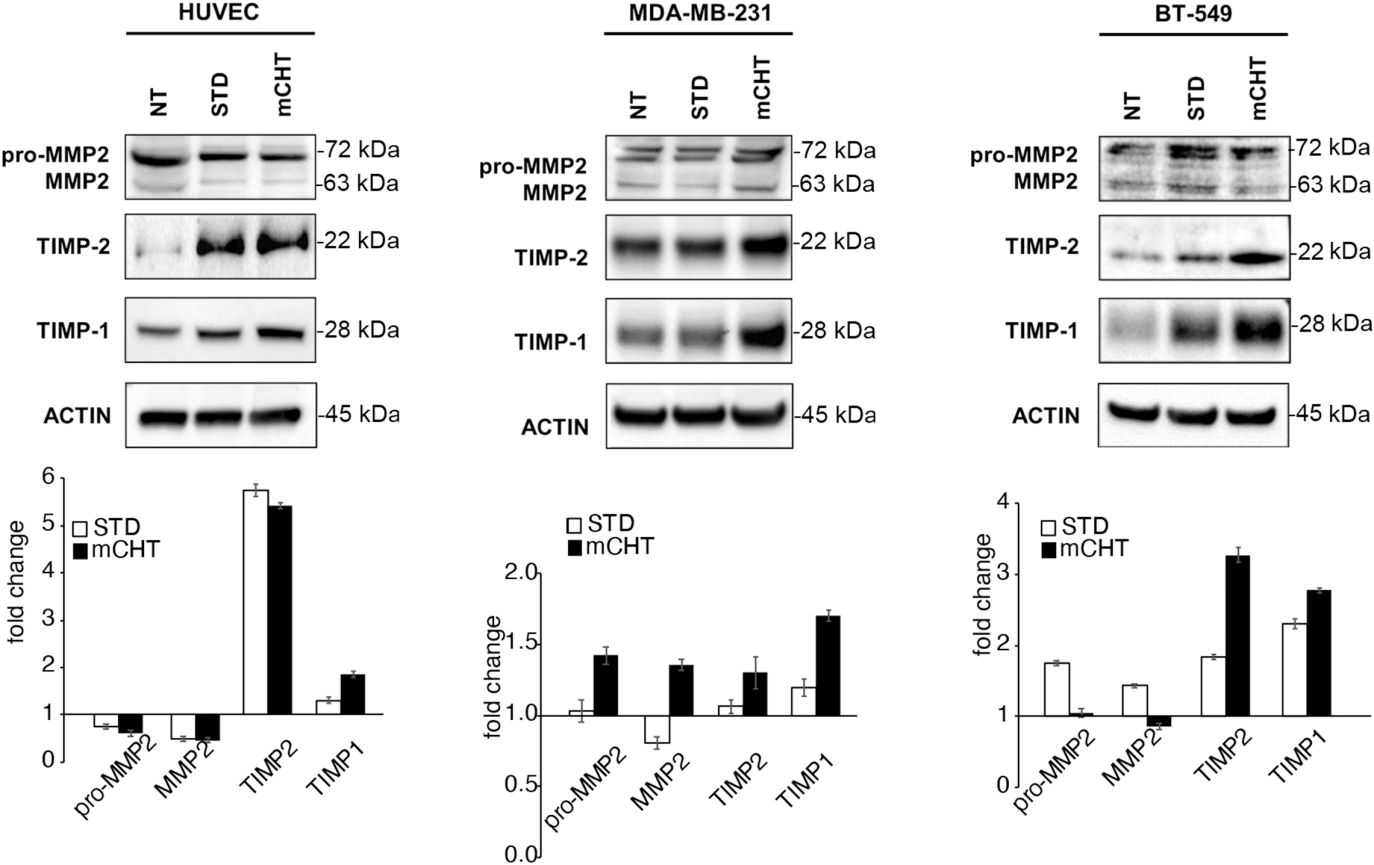
mCHT and STD administration of 5-FU+VNR differently modulate MMPs inhibitors in HUVECs and TNBC cells. On the top: representative western blots of HUVECs, MDA-MB-231 and BT-549 cells treated with mCHT or STD 5-FU+VNR. A representative experiment out of three is shown. On the bottom: protein expression levels as calculated by densitometry of the western blot results. Quantification of protein expression levels was normalized to the loading control actin and compared to the untreated control set as 1. Values represents the average ± SD of three independent experiments.

Then, we analyzed Matrix Metalloproteinase 2 (MMP2), Tissue Inhibitor of Metalloproteinases 1 (TIMP1) and 2 (TIMP2) expression, whose activities are essential for matrix degradation during neo-angiogenesis and metastasis formation (35). In HUVECs, both STD and mCHT administration of 5-FU+VNR similarly reduced MMP-2 expression and strongly upregulated its inhibitor TIMP-2 (**Figure 6**), whereas TIMP-1 levels increased only after mCHT treatment. Instead, in TNBC cells, MMP2 expression was not, or only mildly, affected by either the mCHT or STD treatment and the increase of both inhibitors was not as marked as in HUVECs (**Figure 6**). In particular, the expression of TIMP1 and TIMP2 increased only, or was more upregulated, after mCHT treatment in MDA-MB-231 and BT-549 cells, respectively.

Altogether, our data show that the combination of 5-FU+VNR is always more effective in reducing the migration/invasion of both ECs and TNBC cells when given mCHT *vs* STD. Notably, ECs’ capability of migration/invasion is also affected, even though to a lesser extent, by STD administration of 5-FU+VNR, whereas migratory and invasion activities of TNBC cells are only very mildly affected.

### Metronomic and standard treatments with 5-FU+VNR disrupt FAK/VEGFR2/VEGF-mediated signaling in HUVECs and TNBC cells and elicit different cell death modalities

**Figure 7:**
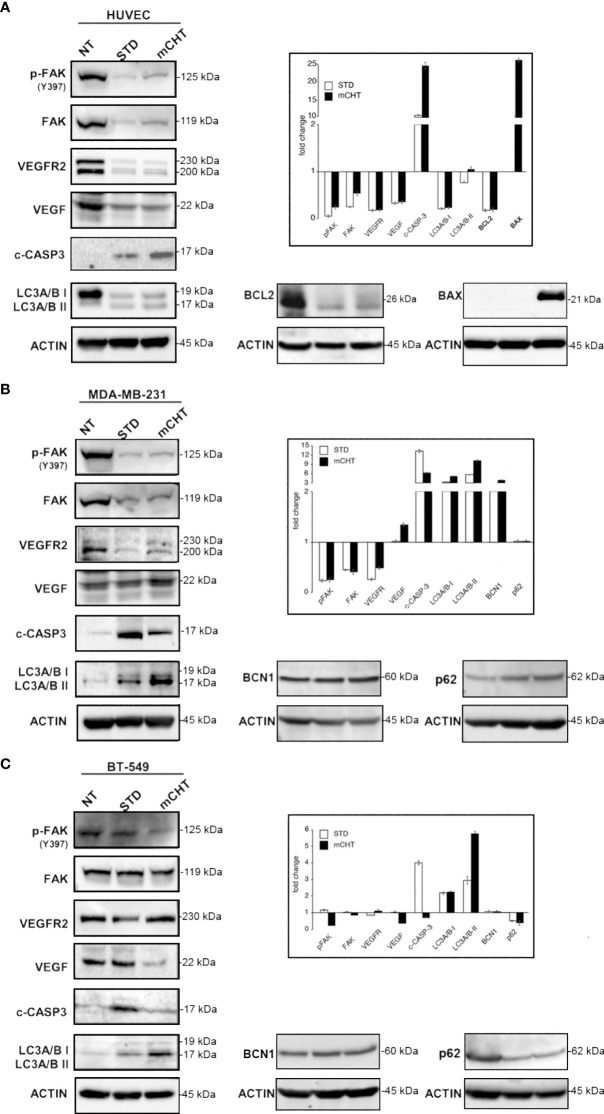
Metronomic and standard treatment with 5-FU+VNR differently affect pathways involved in migration, angiogenesis, apoptosis and autophagy in HUVECs and TNBC cells. Representative western blots of HUVECs, MDA-MB-231 and BT-549 treated with STD or mCHT 5-FU +VNR. In the insets: protein expression levels as calculated by densitometry of the western blot results. Quantification of protein expression levels was normalized to the loading control actin and compared to the untreated control set as 1. Values represent the average ± SD of three independent experiments.

Then, we sought to investigate the molecular mechanisms by which 5-FU+VNR STD and mCHT treatments affect HUVECs and TNBC cells migration and viability. We found that FAK phosphorylation as well as expression levels are strongly reduced by STD and mCHT treatment in both HUVECs and MDA-MB-231 cells (**Figures 7A, B**). On the contrary, in BT-549 cells FAK expression did not significantly change upon the different treatments, albeit its phosphorylation levels were strongly reduced by the mCHT schedule only (**Figure 7C**). FAK is involved in the regulation of angiogenesis *via* the transcription of VEGFR2 and VEGF (11, 36, 37). In agreement with the observed downregulation of FAK expression and activation, in HUVECs and MDA-MB-231 cells both STD and mCHT administration of 5-FU+VNR resulted in a strong reduction of the expression of VEGFR2 (**Figures 7A, B**); however, we observed that the expression of its ligand– VEGF - was downregulated only in HUVECs and not in MDA-MB-231 cells. At variance, in the case of BT-549 cells we found that the receptor was only slightly reduced by both schedules of treatment whereas a strong reduction of the expression of the ligand was evident following mCHT treatment (**Figure 7C**).

Then, we investigated which cell death-related mechanisms were triggered by the different schedules of treatment. In all cell lines caspase-3 was cleaved to a certain extent: whereas cleaved caspase-3 was strongly accumulated only after mCHT treatment in HUVECs (**Figure 7A**), in TNBC cells the opposite occurred i.e., cleaved caspase-3 levels significantly increased only after STD treatment (**Figures 7B, C**), consistently to our previous report where we also investigated BCL2 and BAX modulation upon the two schedules of treatments (31). Hence, to complete the picture we analyzed their levels in treated HUVECs. In accord with the high levels of cleaved caspase-3 a profound depletion of BCL2 and a strong induction of BAX were evident only in mCHT-treated HUVECs (**Figure 7A**). We therefore investigated whether autophagic cell death could be triggered by STD administration. Surprisingly, a strong downregulation of the autophagy marker LC3A/B-I accompanied by a slight processing to LC3A/B-II precursor occurred after both schedules of treatment (**Figure 7A**) suggesting that autophagic death is likely not involved in the loss of viability of HUVECs. Instead, in TNBC cells, both treatments induced LC3A/B-I and its processing to LC3A/B-II but its levels resulted significantly higher only upon mCHT schedule (**Figures 7B, C**), in agreement with our precedent report (31). Accordingly, in MDA-MB-231 cells BCN1 expression was more induced by mCHT than STD schedule whereas no p62(SQSTM1) variations were evident (**Figure 7B**). In BT-549, BCN1 expression remained unchanged upon either treatment and was accompanied by decreased expression of p62 (**Figure 7C**).

Overall, these data show that 5-FU+VNR, either given STD or mCHT, strongly downregulates the VEGF/FAK signaling in ECs and in MDA-MB-231 cells whereas in BT-549 cells this pathway is profoundly affected only by the mCHT schedule. Notably, only mCHT treatment is cytotoxic *via* apoptosis induction in ECs; instead in TNBC cells autophagy induction is predominant over apoptosis induction whereas the opposite occurs upon STD treatment.

### Metronomic treatment with 5-FU+VNR is more effective in disrupting neo-angiogenesis than standard treatment

**Figure 8:**
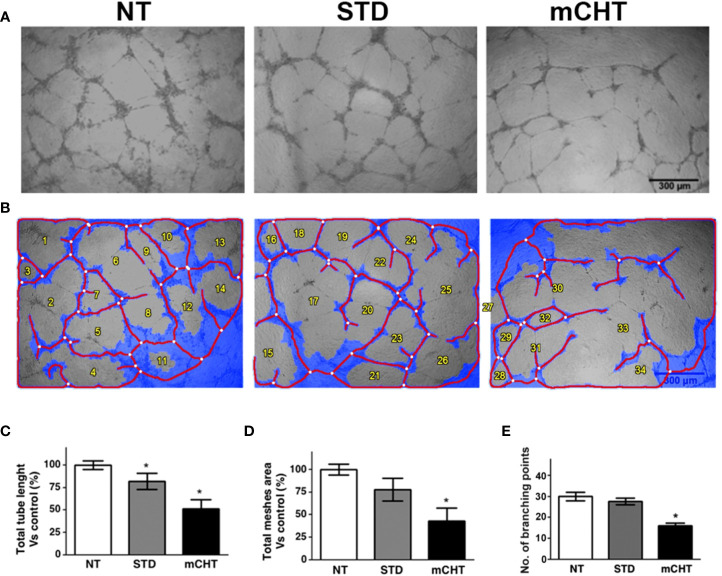
Metronomic administration of 5-FU+VNR is more active than standard treatment in impairing HUVECs neoangiogenesis. **(A)** Representative images of tube formation assays performed on HUVECs after STD or mCHT treatment. **(B)** Processed images by Wimasis software. Total tube length **(C)** and total meshes area **(D)** were quantified by Image J software and graphically represented as a percentage of the software control. Numbers of branching points were measured by Wimasis **(E)**. Blue, the tubular structure. Red, tubes. White, branching points. Values represent the average ± SD of three independent experiments, *p < 0.05.

In the context of tumor growth, ECs migration and survival are crucial, as well as the ability of ECs to form new vessels. To assess whether the 5-FU+VNR treatment can affect also neo-angiogenesis, we performed tube formation assay using HUVECs treated with 5-FU+VNR given STD or mCHT (**Figure 8**). Already within 4h ECs spontaneously initiated vascular morphogenesis and formed multicellular tubular networks in STD and NT control cells, but not in mCHT-treated cells, which begin forming the vascular structure at 8 h remaining lower in number (**Supplementary Figure 2**). The organization of the tubular network continued to be more impaired by the mCHT than the STD treatment up to 24h as demonstrated by the measurement of the total tube length, the total meshes area, and the number of branching points (**Figures 8A–E**). In fact, all these parameters were mildly reduced by STD as compared to control cells. On the contrary, mCHT administration of 5-FU+VNR resulted in 50% reduction of all three measurements compared to control cells. Altogether these findings indicate that the mCHT schedule is more effective in disrupting neo-angiogenesis than the STD schedule.

### Conditioned medium from TNBC cells treated with 5-FU+VNR under mCHT schedule inhibits HUVECs migration and abolishes clonogenic survival

**Figure 9:**
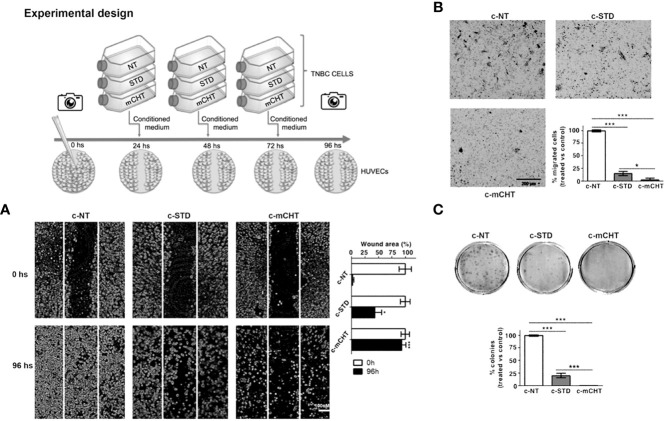
Conditioned medium from metronomically 5-FU+VNR-treated MDA-MB-231 cells suppresses HUVECs migration and colony formation. Schematic drawing of the experimental design. **(A)** Representative images of scratch tests performed on HUVECs before (0h) and 96h after treatment with conditioned medium from MDA-MB-231 cells treated with 5-FU+VNR given STD (c-STD) or mCHT (c-mCHT). Conditioned medium from untreated MDA-MB-231 (c-NT) was used as control. The area of the still open wound after 96h is quantified as a percentage of the initial scratched area. Values represent the average ± SD of three independent experiments, *p <0.05; ***p <0.001. **(B)** Representative images of Transwell assays performed on HUVECs at the end of the treatment with c-STD or c-mCHT media. Migrated cells were stained with crystal violet, counted, and graphically expressed as percentage of untreated control (c-NT). Values represent the average± SD of three independent experiments, *p <0.05; ***p <0.001. **(C)** Representative images of colony formation assay performed on HUVECs 10 days after the end of the treatment with c-STD or c-mCHT media. Colonies were stained with crystal violet and counted. *On the bottom*: number of colonies grown after treatments quantified as a percentage of untreated controls. Values represent the average ± SD of three independent experiments, ***p <0.001.

The interactions between the tumor and its microenvironment are crucial for tumor formation, progression, and the development of metastasis; in particular, the crosstalk between cancer cells and ECs participates in promoting neo-angiogenesis and cell motility (38). Therefore, we investigated whether the conditioned medium harvested from TNBC cells treated with STD (c-STD) or mCHT (c-mCHT) combination of 5-FU+VNR (as described in the experimental scheme of **Figure 9**) might modulate ECs migration and survival.

We found that c-STD-medium from MDA-MB-231 cells significantly affected the migration and clonogenic survival of HUVECs but the effect was much weaker than that exerted by c-mCHT-medium (**Figure 9**). In fact, scratch assays analyzed 96 hours after the incubation with MDA-MB-231-derived c-mCHT- or c-STD medium revealed that 90% *vs* 40% of the initial scratch area was still open (**Figure 9A**). Similarly, MDA-MB-231-derived c-mCHT medium was more effective than c-STD medium in inhibiting HUVECs migration. In this case, TNBC cells-derived c-mCHT medium reduced the percentage of migrated HUVECs to 2.5% of the control compared to the 15% after incubation with c-STD medium (**Figure 9B**). Finally, HUVECs’ clonogenic ability was completely abrogated by MDA-MB-231-derived c-mCHT medium whereas after incubation with c-STD medium 20% of colonies were still able to outgrow (**Figure 9C**).

Overall, these data show that mCHT treatment is more effective than STD in inducing TNBC cells to release factors contributing to suppress migration and survival of ECs.

### Conditioned medium from TNBC cells treated with 5-FU+VNR under mCHT schedule inhibits angiogenesis in Matrix plugs *in vivo*

**Figure 10:**
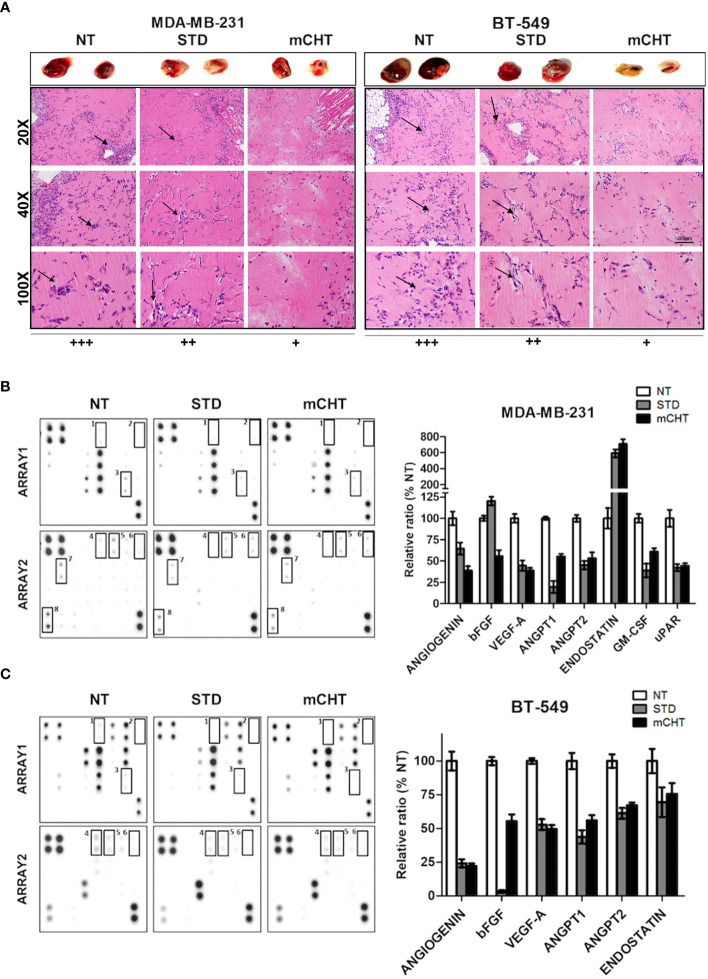
Conditioned medium from TNBC cells treated with 5-FU+VNR under mCHT schedule inhibits angiogenesis *in vivo* and modulate the expression of angiogenesis-related protein. MDA-MB-231 and BT-549 cells’ supernatant collected at the end of a 96h-treatment with the respective IC_50s_ of 5-FU+VNR under the mCHT or STD schedule and from untreated cells was added into Matrigel and injected subcutaneously into C57/BL6 mice. Four days after the injection, mice were sacrificed, and plug vascularization was evaluated. **(A)**, *top*: two representative images of the Matrigel plugs for each treatment are shown. *bottom*: Hematoxylin-eosin staining was used to identify neovessel formation in the Matrigel plugs. Neovessels were evaluated by two different pathologists and scored as +++, ++ or + depending on their frequency. 20X, 40X and 100X, representative images are shown. Arrows indicate infiltrating endothelial cells. **(B, C)** Analysis of the expression profiles of proteins involved in the angiogenesis process. Proteome Array membranes were incubated with c-NT, c-STD and c-mCHT derived from both TNBC cell lines overnight at 4°C as described in the Materials and Methods section. Control proteins are spotted in two opposite corners of the arrays. The experiment was repeated using duplicate conditioned media of the TNBC cells. Modulated proteins in treated vs control cells are highlighted with squares and indicated by numbers. Duplicate spots for each protein are present on the array membrane. On the right of the array membranes: modulated protein quantified as a percentage of untreated controls.

To evaluate the effect of mCHT vs. STD treatment on angiogenesis *in vivo*, we performed the UltiMatrix sponge assay in C57/BL6 female mice (33, 34). Days after the implantation of the plugs supplemented with conditioned medium from MDA-MB-231, BT-549 cells or untreated controls, hemorrhagic lesions were evaluated in the Matrigel pellets (**Figure 10A**, top). Visual examination of the pellets indicated that both c-mCHT- and c-STD-medium from treated MDA-MB-231 and BT-549 cells inhibited the Matrigel sponges’ vascularization compared to NT. Notably, BT-549-derived c-mCHT-medium was remarkably more efficacious than c-STD-medium in preventing angiogenesis of the Matrigel sponges, whereas c-mCHT and c-STD media derived from MDA-MB-231 cells reduced angiogenesis to a similar extent and were less effective compared to BT-549-derived c-mCHT-medium. Given the remarkable difference between c-mCHT- and c-STD-medium from treated BT-549 cells in affecting angiogenesis *in vivo*, quantification of vascularization - evaluated by measuring hemoglobin content in the sponges - was performed. The results confirmed that BT-549-derived c-mCHT-medium significantly inhibited angiogenesis *in vivo* and was much more effective compared to c-STD-medium (**Supplementary Figure S3**). These differences were also confirmed by histological analysis of the plugs supplemented with the different conditioned medium derived from both cell lines. Neovessel formation in the plugs supplemented with c-NT supernatants derived from both cell lines was evaluated as (+++). Both MDA-MB-231 cells-derived c-STD- and c-mCHT-medium similarly reduced the vascular density in Matrigel plugs (++). At variance, rare microvascular formation was observed in sponges supplemented with c-mCHT (+) compared to c-STD (+++) supernatants from BT-549 cells (**Figure 10A**, bottom and **Supplementary Figure S3**).

Overall, inhibition in the growth of new blood vessels and a very strong reduction in hemoglobin content was observed, which was more pronounced when c-mCHT-derived supernatants were used *vs* c-STD-derived ones.

### Conditioned medium from TNBC cells treated with 5-FU+VNR under mCHT treatment modulates the expression of angiogenesis-associated factors

To further investigate the effect of c-mCHT on angiogenesis-related proteins - with either pro- or antiangiogenic function- we collected supernatants from MDA-MB-231 and BT-549 cells untreated or treated with the mCHT and STD schedules and analyzed them through a proteome profiler human angiogenesis antibody array (**Figures 10B, C**). In both TNBC cell lines-derived c-mCHT- and c-STD-medium the downregulation of pro-angiogenic factors, (angiogenin, bFGF, VEGF-A, angiopoietin-1, angiopoietin-2) was observed. In addition, in c-mCHT- and c-STD-medium from MDA-MB-231 cells also downregulated were the Granulocyte Macrophage-Colony Stimulating Factor (GM-CSF) - implicated in cell cytoskeleton rearrangement promotion and macrophages recruitment in TNBC cells (39)- and the glycolipid-anchored receptor (uPAR) - involved in cell adhesion and migration (40). In the same conditioned media also an upregulation of endostatin - a known inhibitor of endothelial cell proliferation, migration, and angiogenesis (41)- was observed. At variance, a modest downregulation of the same protein was detectable in c-mCHT- and c-STD-medium from BT-549 cells.

## Discussion

Despite advances in cancer treatment, metastases remain the main cause of death in most cancer patients, including TNBC ones (42–44). TNBC is one of the most aggressive tumors (45), and the standard treatment with chemotherapy usually does not block metastasis formation. Indeed, among more than 200 FDA-approved drugs, very few exhibit anti-metastatic activity (46), and this effect is evident only when patients are treated using a metronomic protocol (25). Metastasis formation is a complex process, requiring the formation of new blood vessels through which metastatic cancer cells spread to other anatomic sites (47). Thus, to understand the anti-metastatic effect observed in the clinic we investigated *in vitro* and *in vivo* the effects of mCHT combination of 5-FU and VNR on HUVECs and TNBC cell migration compared to the STD treatment.

First, we demonstrate that 5-FU and VNR given mCHT as single agents, or in combination, strongly reduce the survival of ECs, as we previously reported for TNBC cells (31). Importantly, this effect was achieved using doses that are about 100-fold lower than those given STD (**Figure 1**). In addition, the IC_50_ of 5-FU+VNR was similar in both HUVECs and TNBC cells when given mCHT, differently from what observed when the combination of drugs was given as STD: in this case, a 3-to-4-fold higher dose of 5-FU was needed to kill the 50% of TNBC cells *vs.* HUVECs (**Table 1**). The strong inhibitory effect on both tumor cells and ECs using a much lower amount of the drugs, accounts for the reduction of the toxic side effects observed for mCHT, compared to the STD regimen, as reported in several clinical trials (27, 28, 48, 49). Moreover, our data - showing that the combination of 5-FU+VNR given mCHT is active on both HUVECs and TNBC cells within the same range of doses - suggest that this protocol is, simultaneously, anti-tumoral and anti-angiogenic. These findings are particularly relevant for the clinical practice since they indicate that using a metronomic combination of 5-FU and VNR both tumor and vascular endothelial cells are targeted, thus delivering a double hit to the tumor. Our data are in line with the literature, which defines metronomic chemotherapy as a therapy simultaneously targeting tumor, endothelial, and immune system cells (50).

Importantly, we observed that both HUVECs and TNBC cells retain clonogenic capability following STD treatment. On the contrary, re-growth was completely abolished by the mCHT treatment (**Figure 2**), suggesting a cytostatic *vs.* a cytotoxic effect of the STD and mCHT protocols, respectively. These data support and explain the effects observed in the clinical setting (51), where relapses occur more frequently, following the STD *vs* mCHT protocol. In fact, a better control of recurrences and metastasis has been observed after mCHT and significantly long periods of clinical benefit (Complete + Partial + Stable Disease ≥ 24 weeks) have been reported (52, 53). This interpretation is further supported by the migration and invasion assay (**Figures 3**–**5**). We observed that only the mCHT combination of 5-FU+VNR strongly inhibited cell motility of both ECs and TNBC cells. When administered as STD, 5-FU+VNR significantly reduced the ability of migration and invasion of ECs but not TNBC cells. Accordingly, in the tube formation assay, the mCHT administration resulted in ~50% of the total tube length, the total mesh area and the numbers of branching points compared to ~20% observed after STD administration (**Figure 8**; **Supplementary Figure 2**). Several studies reported an anti-migratory effect of some antitumoral agents when given metronomically, such as ceramide analogs, docetaxel, the 5-FU prodrug UFT (uracil plus tegafur) plus cyclophosphamide (54–56).

Tissue inhibitor of metalloproteases 1 and 2 (TIMP-1 and-2) play an inhibitor role in cell migration and neo-angiogenesis by blocking the matrix degradation activity of several metalloproteases (MMPs). In particular, it has been shown that TIMPs negatively regulate angiogenesis by inhibiting the formation of new vessels (35). Accordingly, our data suggest that 5-FU+VNR can affect neo-angiogenesis *via* modulations of TIMP-1 and TIMP-2. Specifically, in HUVECs, TIMP-2 levels were strongly upregulated after STD and mCHT administration, whereas TIMP-1 was induced only by mCHT treatment. Instead, TNBC cells significantly upregulated both TIMP-1 and TIMP-2 expression only under the mCHT protocol (**Figure 6**). Given that both proteins can be secreted, it is tempting to speculate that mCHT-treated TNBC cells actively participate in modulating the remodeling processes of ECM required for both migration and neo-angiogenesis. These results, together with the lack of closure of the wound (**Figure 3A**) and the induction of apoptotic markers (**Figure 7**), indicate that besides an anti-migratory effect a cytotoxic effect is also triggered by mCHT treatment on HUVECs.

FAK’s high expression and phosphorylation levels are associated with cancer progression and metastasis by promoting tumor and endothelial proliferation and migration (57). In particular, FAK also promotes neo-angiogenesis by upregulating pro-angiogenic factors, such as VEGFR2 and VEGF (11, 36, 37). Very recently Shiau and colleagues reported a positive association between FAK and VEGFR2 in TNBC patients and demonstrated that FAK knockdown inhibited endothelial tube formation in a zebrafish model; in addition, they also showed in a mice xenograft model that FAK inhibitors could suppress tumor growth and tumor vascular formation *via* VEGFR2 and VEGF downregulation (58). These data are in line with our results: in fact, we observed that the combination 5-FU+VNR strongly suppressed the levels of total and active FAK as well as VEGFR2 in both HUVECs and MDA-MB-231, regardless of the modality of administration (**Figure 7**). Interestingly, in BT-549 -having a different mutational background compared to MDA-MB-231 (**Supplementary Table S1**)- only the mCHT schedule significantly reduced p-FAK and VEGF levels. Despite an impairment of the FAK/VEGFR axis after both schedules of treatments, (**Figure 7**) MDA-MB-231 cells were still able to migrate and overspread the matrix following STD administration of 5-FU+VNR whereas a very significant decrease of cells able to migrate and invade the matrix occurred after mCHT treatment (**Figures 3**–**5**). Notably, BT-549 cells’ capability to migrate and invade was affected by both schedules (**Figures 3**–**5**), even though only mCHT impairs the FAK/VEGF axis (**Figure 7**). These findings suggest that the different genetic backgrounds of the two TNBC cell lines might result in the modulation of different pathways and thus influence the response to the diverse schedules of treatment. Despite this, in both TNBC and endothelial cells, only mCHT exerts a striking anti-angiogenic, anti-migratory and anti-invasion effect, thus indicating that additional factors remain to be identified to further clarify the molecular basis of this effect.

Given the reduction in cell survival recorded by MTT and clonogenic assays (**Figures 1**, **2**) and the visible reduction in cell density observed in the scratch assay (**Figure 3**) we aimed at defining the mechanisms of cytotoxicity triggered by mCHT vs STD schedule in HUVECs. Interestingly we found that, despite a profound depletion of anti-apoptotic BCL2 triggered by both schedules of treatment, only mCHT strongly upregulated pro-apoptotic BAX which was accompanied by the accumulation of cleaved caspase-3 (**Figure 7A**), thus indicating that mCHT-induced cytotoxicity in HUVEC cell is *via* apoptosis. As far as STD-induced cytotoxicity we cannot exclude that apoptosis played a role since some caspase 3 was detectable in the cleaved form and BCL2 levels dropped abruptly; however, we did not find any BAX induction. Checking other possible modalities of cell death we observed that both mCHT and STD treatments, despite slightly increasing the conversion of the autophagic marker LC3A/B-I to LC3A/B-II (59), strongly suppressed its overall expression thus suggesting that autophagic death is likely not involved in the loss of viability of HUVECs. Several evidence point to an involvement of a sustained and active autophagic flux for endothelial cell differentiation and normal physiology. In fact, autophagy is required for the development of vascular ECs and its appropriate regulation is pivotal during fundamental adaptive responses such as cell proliferation and other endothelium functions (60, 61). Given that only upon mCHT schedule we observed a strong suppression of LC3A/B-I accompanied by marked activation of caspase-3 we might conclude that a treatment directed to impair autophagy is able to favor the apoptotic response even when low doses of drugs are used.

At variance, a stronger induction of the expression and conversion of LC3A/B-I to LC3A/B-II (**Figure 7A**), accompanied by reduced levels of active caspase-3, was evident in mCHT- vs STD-treated TNBC cells, thus confirming our previous data indicative of autophagic cell death (31). To strengthen these data, we also analyzed the expression of other actors involved in effecting the autophagic process namely BCN1 and p62/SQSTM1 (59). In MDA-MB-231 BCN1 was induced more by mCHT than the STD treatment whereas p62 expression did not change (**Figure 7B**). The opposite trend was observed in BT-549 cells where BCN1 expression remained unvaried and p62 levels were reduced (**Figure 7C**). Our data are not in agreement with the most common model of active autophagic flux - where an upregulation of BCN1 is followed by an increase of the processing of LC3A/B-I to LC3A/B-II and a degradation of p62 (59) - since they suggest that the autophagic flux is interrupted. However, as uncovered by the several studies appeared in the literature, it is still unclear whether cell death ensues by the failure of the autophagic flux or it is actively regulated by autophagic factors (59). Moreover, since autophagy has also been associated with inhibition of cell migration in different tumors (62), we cannot exclude this process’s involvement in the anti-migratory effect seen in TNBC cells after mCHT administration of 5-FU+VNR.

Cancer development is not only directly promoted by tumor cells but also *via* interaction with microenvironmental cellular and molecular elements, which in turn strongly influences tumor progression and metastasis formation and the subsequent clinical outcome (63). Therefore, we studied whether STD or mCHT treated-TNBC cells influence *via* paracrine activity HUVECs migration and colony formation ability (64–66). In this experimental setting, mCHT appeared to be significantly more efficient than STD protocol in inhibiting ECs migration and survival *in vitro* (**Figure 9**): in fact, only conditioned medium from mCHT-treated TNBC cells completely suppressed transwell migration (**Figure 9B**) and colony formation (**Figure 9C**). Indeed, neo-angiogenesis was strongly impaired in the *in vivo* model using medium from treated *vs* untreated TNBC cells. Notably, the effect was more marked when using medium from mCHT-treated *vs* STD-treated TNBC cells (**Figure 10A**).

Collectively, these results may explain a major effect observed in treating patients with STD therapy, i.e., the regenerative capability of damaged tumor vasculature after treatment, despite high doses of drugs employed (67). Remarkably, our findings also contribute to understanding the mechanism underlying the effectiveness of the mCHT schedule in disease control. In fact, our results indicate that the combination of 5-FU+VNR acts on ECs directly and *via* factors released from treated TNBC cells. Indeed, the results obtained by proteomic profiling of the factors released by STD- and mCHT-treated TNBC cells revealed that treatments modulated the expression of several angiogenesis-related proteins (**Figures 10B, C**).

In summary, we show that the combination of 5-FU+VNR administered mCHT *in vitro* is more effective in simultaneously inhibiting ECs and TNBC cell migration/invasion and re-growth than the STD schedule of treatment. Moreover, we confirmed the anti-angiogenic effect of the mCHT protocol in an *in vivo* system. Therefore, our pre-clinical data offer a way to interpret how the therapeutic effect of the metronomic administration of 5-FU plus VNR (68) is reached, i.e., by targeting both TNBC and endothelial cells. In particular, our findings that only mCHT completely blocks colony re-growth and affects both ECs and TNBC cells migration/invasion, tube formation, and new vessel formation *in vivo*, strongly indicate that the stabilization of tumor growth observed in TNBC patients treated with mCHT is likely due not only to direct cytotoxic effects but also to anti-metastatic and anti-angiogenic effects.

Taken together with other published data, our results confirm the multimodality mechanism of action of mCHT and contribute to understanding of the cellular and molecular mechanisms underlying the effect observed in clinical trials in TNBC patients treated with mCHT therapy (69). Moreover, our findings support the rationale for its employment in TNBC patients, where the dual targeting of the tumor and its vasculature at the same time would result in better therapeutic outcomes. Further confirmations in the clinical setting are urgently needed through randomized trials to assess the role of mCHT in the treatment’s algorithm of TNBC patients. Additionally, even though these data are limited to the TNBC model, inhibition of angiogenesis and block of migration should represent relevant endpoints to be assessed in different subtypes of breast cancer, i.e., HR+ after cell-cycle inhibitors (CDK 4/6) or in those tumors characterized by the loss of endocrine-sensitivity (70).

## Data availability statement

The original contributions presented in the study are included in the article/**Supplementary Material**. Further inquiries can be directed to the corresponding author.

## Ethics statement

All the *in vivo* procedures applied were approved by the local animal experimentation ethics committee (ID# #06_16 Noonan) of the University of Insubria and by the Health Ministry (ID#225/2017-PR).

## Author contributions

AS wrote the first draft of the manuscript and with LC, designed, performed experiments and analyzed data. MDG, AI, NC, MG, AB, GP performed experiments and analyzed data. AA analyzed data. MEC and ML, provided critical revision of the manuscript EG. analyzed data and provided critical revision of the manuscript. MGC conceived the research, supervised the research, analyzed data and provided critical revision of the manuscript. All authors contributed to the article and approved the submitted version.

## Funding

This work was supported by A&Q- Polo per la Qualificazione del Sistema Agro-Industriale per indagini di laboratorio collegate allo studio pre-clinico *in vitro* VICTOR-9 to MGC; by Horizon 2020 Instand-NGS4P number: 874719 and by a grant of MIUR, PRIN 2017 to ML; by the Italian Ministry of Health Ricerca Corrente-IRCCS MultiMedica, and by Italian Association for Cancer Research (AIRC-MFAG, ID 22818) and a research grant funded by the Cariplo Foundation (ID 2019-1609) to AB.

## Conflict of interest

The authors declare that the research was conducted in the absence of any commercial or financial relationships that could be construed as a potential conflict of interest.

## Publisher’s note

All claims expressed in this article are solely those of the authors and do not necessarily represent those of their affiliated organizations, or those of the publisher, the editors and the reviewers. Any product that may be evaluated in this article, or claim that may be made by its manufacturer, is not guaranteed or endorsed by the publisher.
