# Supplementary material for: Co-targeting triple-negative breast cancer cells and endothelial cells by metronomic chemotherapy inhibits cell regrowth and migration via downregulation of the FAK/VEGFR2/VEGF axis and autophagy/apoptosis activation
Source: Front Oncol. 2022 Nov 30;12:998274. doi: 10.3389/fonc.2022.998274 (PMC9749857; doi:10.3389/fonc.2022.998274)
Supplement: Supplementary file 1 [file DataSheet_1.zip › Data Sheet 1.PDF]

**SUPPLEMENTARY MATERIAL**

## Supplementary Figure S1

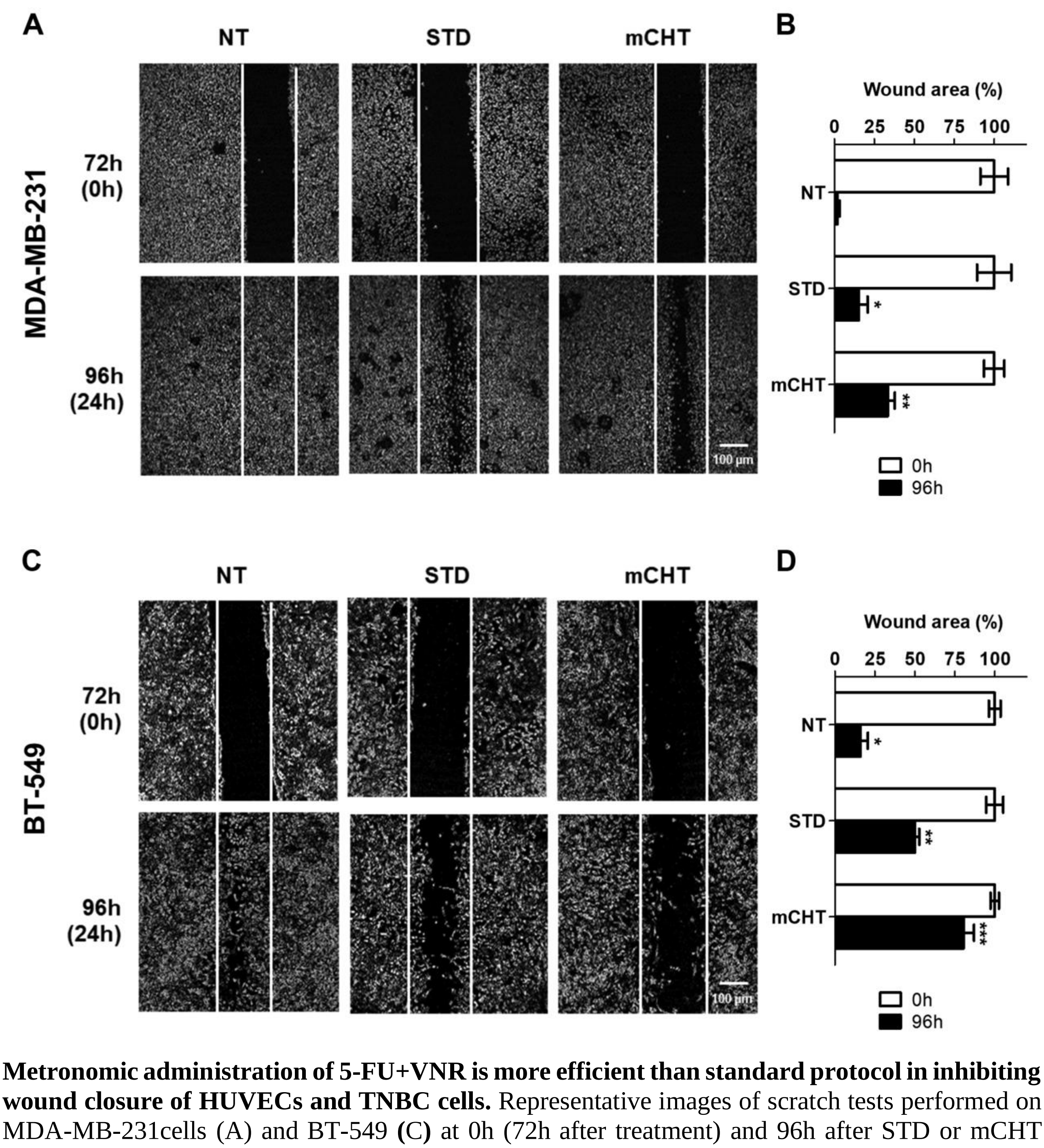


**Metronomic administration of 5-FU+VNR is more efficient than standard protocol in inhibiting wound closure of HUVECs and TNBC cells.** Representative images of scratch tests performed on MDA-MB-231cells (A) and BT-549 **(C)** at 0h (72h after treatment) and 96h after STD or mCHT treatment with 5-FU+VNR. The area of the still open wound after 96h is quantified as a percentage of the initial scratch (B, D). Values represents the average ± SD of three independent experiments, ***p <0.001.

## Supplementary Figure 2

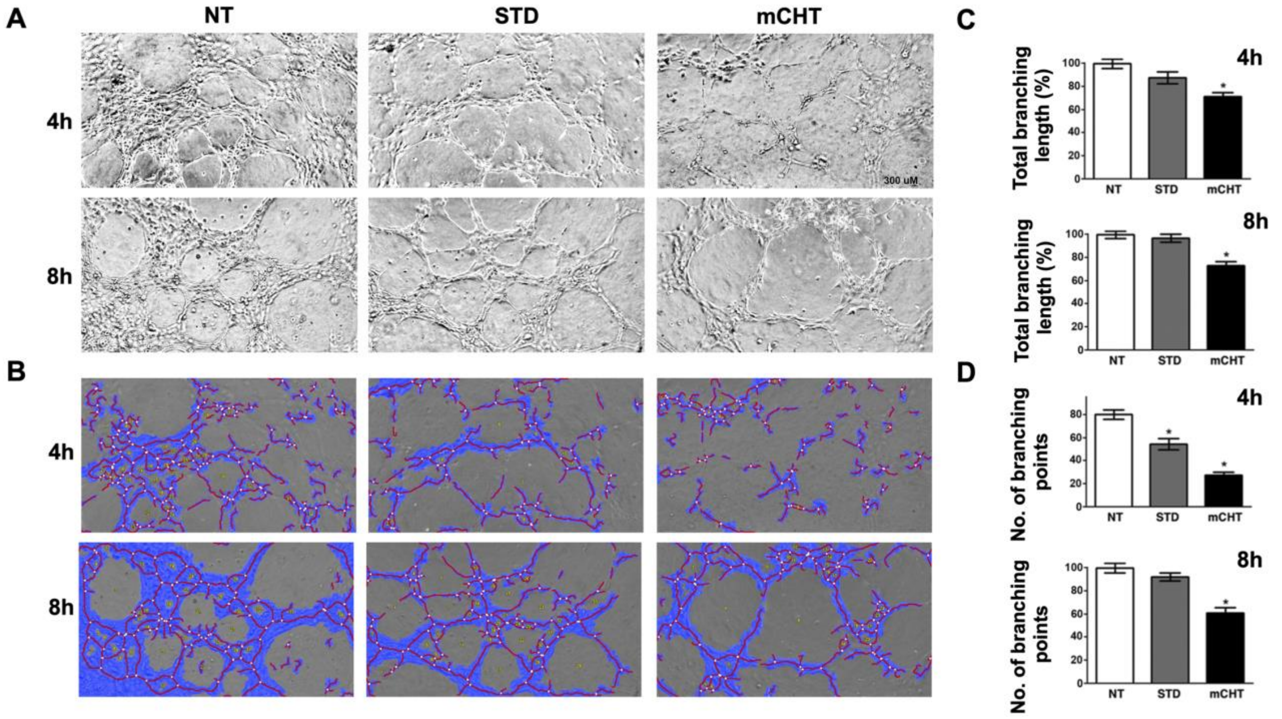


**Metronomic administration of 5-FU+VNR is more effective than standard treatment in impairing HUVECs neoangiogenesis.** (A) Representative images of tube formation assays, performed at 4h and 8h, on HUVECs after STD or mCHT treatment. (B) Processed images by Wimasis. Total branching length (C) were quantified by Image J software and graphically represented as a percentage of the untreated control. Numbers of branching points were measured by Wimasis (D). Blue, the tubular structure. Red, tubes. White, branching points. Values represent the average ± SD of three independent experiments, *p <0.05.

## Supplementary Figure S3

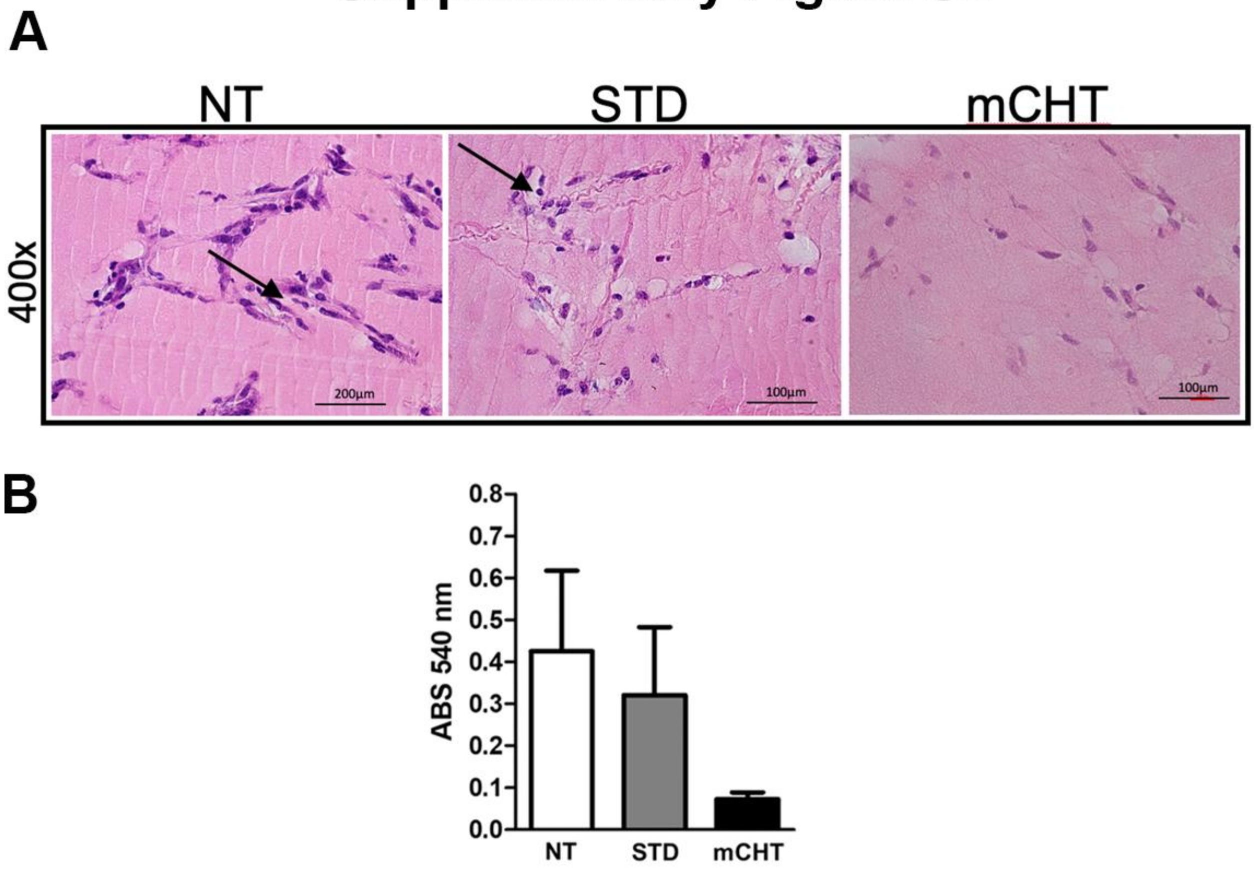


**Conditioned medium from BT-549 cells treated with 5-FU+VNR under mCHT schedule inhibits angiogenesis *in vivo*.** A. Hystological images of BT-549. Magnification: 400X. Arrows indicate infiltration endothelial cells. B. BT-549 plugs were minced and diluted in water to measure the hemoglobin content with a Drabkin's Reagent Kit.

**Supplementary Table S1**

### Supplementary Table 1

| | DNA damage/repair pathways | | | RAS/MAPK pathway | PI3K pathway |
|---|---|---|---|---|---|
| **MDA-MB-231** | ATM ATRX | BRCA1 | p53 | BRAF, KRAS; NF1; NF2, MAP2K4 | |
| **BT-549** | ATR CHEK2 | BRCA1 | p53 | | PTEN |

Genetic background of the TNBC cell lines used in the paper according to the Cosmic database (https://cancer.sanger.ac.uk)
